# Shear Stress Triggers Angiogenesis of Late Endothelial Progenitor Cells via the PTEN/Akt/GTPCH/BH4 Pathway

**DOI:** 10.1155/2020/5939530

**Published:** 2020-04-30

**Authors:** Shao-Hong Wu, Feng Zhang, Shun Yao, Lu Tang, Hai-Tao Zeng, Ling-Ping Zhu, Zhen Yang

**Affiliations:** ^1^Department of Ultrasound, The First Affiliated Hospital, Sun Yat-Sen University, 58 2nd Zhongshan Road, Guangzhou, China 510080; ^2^Department of Cardiovascular Medicine, Xiangya Hospital, Central South University, 87 Xiangya Road, Changsha, China 410008; ^3^Department of Cardiovascular Institute, Guangdong Provincial People's Hospital, Guangdong Academy of Medical Science, Guangzhou, China 510080; ^4^Department of Geriatric Medicine, Xiangya Hospital, Central South University, 87 Xiangya Road, Changsha, China 410008; ^5^Center for Reproductive Medicine, The Sixth Affiliated Hospital, Sun Yat-Sen University, 58, 2nd Village Cross Road, Guangzhou, China 510080; ^6^Division of Emergency Medicine, Department of General Internal Medicine, Department of Emergency Intensive Care Unit & Department of Cardiology & Key Laboratory on Assisted Circulation, Ministry of Health, The First Affiliated Hospital, Sun Yet-Sen University, 58 2nd Zhongshan Road, Guangzhou, China 510080

## Abstract

**Background:**

Shear stress is an effective modulator of endothelial progenitor cells (EPCs) and has been suggested to play an important role in angiogenesis. The phosphatase and tensin homolog (PTEN)/Akt and guanosine triphosphate cyclohydrolase (GTPCH)/tetrahydrobiopterin (BH4) pathways regulate the function of early EPCs. However, the role of these pathways in the shear stress-induced angiogenesis of late EPCs remains poorly understood. Therefore, we aim to investigate whether shear stress could upregulate the angiogenesis capacity of late EPCs and to further explore the possible underlying mechanisms.

**Methods:**

Late EPCs were subjected to laminar shear stress (LSS), and their *in vitro* migration, proliferation, and tube formation capacity were determined. In addition, the *in vivo* angiogenesis capacity was explored, along with the expression of molecules involved in the PTEN/Akt and GTPCH/BH4 pathways.

**Results:**

LSS elevated the *in vitro* activities of late EPCs, which were accompanied by downregulated PTEN expression, accelerated Akt phosphorylation, and GTPCH/BH4 pathway activation (all *P* < 0.05). Following Akt inhibition, LSS-induced upregulated GTPCH expression, BH4, and NO level of EPCs were suppressed. LSS significantly improved the migration, proliferation, and tube formation ability (15 dyn/cm^2^ LSS vs. stationary: 72.2 ± 5.5 vs. 47.3 ± 7.3, 0.517 ± 0.05 vs. 0.367 ± 0.038, and 1.664 ± 0.315 vs. 1 ± 0, respectively; all *P* < 0.05) along with the *in vivo* angiogenesis capacity of late EPCs, contributing to the recovery of limb ischemia. These effects were also blocked by Akt inhibition or GTPCH knockdown (*P* < 0.05, respectively).

**Conclusions:**

This study provides the first evidence that shear stress triggers angiogenesis in late EPCs via the PTEN/Akt/GTPCH/BH4 pathway, providing a potential nonpharmacologic therapeutic strategy for promoting angiogenesis in ischemia-related diseases.

## 1. Background

Tissue ischemia and hypoxia induced by vascular disease are an important pathophysiological mechanism of ischemic disease. Thus, achieving an improved angiogenesis response to tissue ischemia is an effective therapeutic strategy to reduce organ damage in ischemia diseases [1, 2]. Accumulating evidence suggests that adult angiogenesis is not solely the result of endothelial cell (EC) proliferation but also related to the neovascularization function of circulating endothelial progenitor cells (EPCs) [3–6]. In addition, at least two different types of EPCs, early EPCs and late EPCs (or outgrowth EPCs), were recently identified in an *in vitro* cell culture system [7] with distinct cellular properties and biological functions. Early EPCs emerge during the early culture period within 4–7 days, with faint positive staining of VE-cadherin and KDR, low proliferation potential, and strong cytokine release, and are mainly involved in the repair of the injured vascular endothelium. By contrast, late EPCs (or outgrowth EPCs) emerge in the late culture period at up to 2–4 weeks and show stronger expression of VE-cadherin, KDR, and vWF, with high competency to produce endogenous nitric oxide (NO) and enhanced neovasculogenesis. Moreover, the proliferation, migration, and adhesion of late EPCs are promoted by the hypoxia in ischemic tissues, resulting in improved tubular formation ability and enhanced EPC-related angiogenesis to exacerbate the condition [7–9]. Therefore, gaining a better understanding of the mechanisms underlying angiogenesis derived from late EPCs could provide a basis for a novel therapeutic strategy for ischemia diseases.

It is now well established that shear stress has a beneficial effect on homeostasis of the vascular endothelium and also acts as the key trigger for new vessel formation [10, 11], and the beneficial effects of shear stress on ECs and EPCs are mediated exclusively by laminar shear stress (LSS), not turbulent/oscillatory flow [12–16]. In line with previous investigations, we found that LSS increased the migratory, adhesive, and proliferative activities of human early EPCs, which were accompanied by the upregulated expression of tissue type plasminogen activator and enhanced levels of endothelial nitric oxide synthase (eNOS) and superoxide dismutase [17–23]. Thus, LSS is an important nonpharmacological means of modulating the function of EPCs. However, studies on the individual effect of LSS on late EPCs and their angiogenesis capacity are limited.

The tumor suppressor phosphatase and tensin homolog (PTEN), an endogenous inhibitor of the PI3K/Akt/eNOS pathway, constitutes a major determinant of neovascularization at ischemic sites and has been shown to be associated with the angiogenesis functions of ECs and EPCs [24–26]. Hamada et al. revealed that PTEN deficiency in ECs accelerates tumor growth by promoting angiogenesis [24], and Koide et al. found that apoptosis regulator through modulating IAP expression (ARIA) increases membrane-associated PTEN expression while its knockdown leads to an opposite effect, therefore amplifying PI3K/Akt signaling, identifying that ARIA enhances PTEN activation and consequently reduces the PI3K/Akt signaling in ECs and EPCs, leading to the negative regulation in angiogenesis and vasculogenesis [26]. Those studies suggested from different perspectives that PTEN plays as a negative regulator of neovascularization in regard to its expression in ECs or EPCs. In addition, the PTEN/Akt signaling pathway plays important roles in multiple biological processes such as cell proliferation and growth and participates in prototypical endothelial functions such as the regulation of vascular tone, angiogenesis, control of adhesion, and recruitment of leukocytes to the vessel wall [27, 28]. And another study established that the expression and activity of GTP cyclohydrolase I (GTPCH I) and biosynthesis of tetrahydrobiopterin (BH4) were upregulated in human EPCs by treatment with a selective PPAR*δ* agonist GW501516, which downregulated PTEN and thereby increased Akt phosphorylation and consequently enhanced the in vivo regenerative capacity of the EPCs [29].

Our previous study demonstrated that reduced GTPCH expression and decreased BH4 level contribute to impaired early EPCs in a condition of hypertension, and LSS upregulates GTPCH/BH4 pathway by activating sGC/cGMP and suppressing TSP-1 expression, thus enhancing reendothelialization capacity of early EPCs [23]. Considering the similarity between early and outgrowth EPCs, we hypothesized that shear stress may also regulate the expression of the PTEN/Akt and GTPCH/BH4 signaling pathways in late EPCs, which could further enhance their *in vitro* activity and *in vivo* angiogenesis capacity. To test these hypotheses, in the present study, we exposed late EPCs to LSS and examined their *in vitro* function and *in vivo* angiogenesis capacity. We further investigated the role of the PTEN/Akt and GTPCH/BH4 pathways in LSS-mediated alterations of the angiogenesis capacity of late EPCs in a hindlimb ischemia murine model. These findings may provide a novel therapeutic approach to restore the deteriorated EPC function in ischemic disease.

## 2. Material and Methods

### 2.1. Cell Culture and Identification of Late EPCs

This study was approved by the Ethics Committee of Xiangya Medical School of Central South University. Blood samples were obtained from volunteers after receiving informed consent, and late EPCs were isolated and cultured according to our previously reported method [30].

### 2.2. EPC Migration, Proliferation, and Tube Formation *In Vitro*

EPC migration was determined using a modified Boyden chamber placed in a 24-well culture dish containing 500 ml EBM-2 and supplemented with vascular endothelial growth factor (VEGF). Proliferation was determined by a 3-(4,5-dimethylthiazol-2-yl)-2,5-diphenyltetrazolium bromide (MTT) assay, and capillary tube formation was measured as previously reported [17]. Matrigel was added to a 24-well plate to form collagen gels, which were then overlaid with 100 *μ*l cultured EPCs resuspended to 10^6^ cells/ml and incubated at 37°C in an atmosphere of 5% CO_2_. Images of the gel were captured under a phase-contrast microscope. The total length of the tube-like structures in each image was measured and further normalized to the control group and presented into relative tube length according to previous research reports [31, 32].

### 2.3. Laminar Shear Stress Assay

The EPCs were exposed to laminar shear stress with a flow chamber loading device as previously described [18, 23, 30]. The seeded cells were exposed to 5, 15, and 25 dyn/cm^2^ laminar shear stress for up to 15 h or to 15 dyn/cm^2^ laminar shear stress for 5, 10, and 15 h, respectively. The EPCs in the control group were maintained in a static condition. All experiments were performed at 37°C in a CO_2_ incubator.

### 2.4. GTPCH I Knockdown and Pharmacologic Inhibition of Akt

Shear stress experiments were also performed with cells after GTPCH I knockdown by viral transduction using mission shRNA lentiviral transduction particles according to the manufacturer instructions (30MOI, Sunbio Medical Biotechnology Co., Ltd., Shanghai, China) [23]. The target sequence of GTPCH-I was 5′-CcggGCCGCTACCTACTAATGAATTCAAGAGATTCA-TTAGTAGGTAGCGGCTTTTTTg-3′, and the target sequence of the negative control was 5′-CcggTTCTCCGAACGTGTCACGTTTCAAGAGAACGTGACACGTTCGGAGAATTTTTg-3′. After transduction, the cells were washed with phosphate-buffered saline (PBS) and incubated with EPC medium for 48 h; the effect of shRNA transduction on reducing the GTPCH I expression level was confirmed by polymerase chain reaction (PCR).

In addition, the cultured EPCs were preincubated with 10 *μ*mol of the Akt phosphorylation inhibitor LY 294002 (Calbiochem) for 1 h before shear stress stimulation as described previously [18].

### 2.5. Western Blot Analysis

The cells were washed with PBS twice, and total EPC proteins were harvested by cell lysis buffer (#9803, Cell Signaling Technology Inc., Danvers, MA, USA). Protein extracts were subjected to 12% SDS-PAGE and transferred to polyvinylidene fluoride membranes (Immobilon-P, Merck-Millipore, Darmstadt, Germany). The following antibodies were used for western blot analysis: rabbit anti-PTEN (1 : 1000; #9559, Cell Signaling Technology Inc., Danvers, MA, USA), rabbit anti-phosphorylated Akt (Ser473) (1 : 1000; #9271, Cell Signaling Technology Inc., Danvers, MA, USA), rabbit anti-total Akt antibody (1 : 1000; #9272, Cell Signaling Technology Inc., Danvers, MA, USA), mouse anti-GTPCH I (1 : 1000; sc-271482, Santa Cruz Biotechnology, Santa Cruz, CA, USA), and rabbit anti-*β*-actin (1 : 1000; #4970, Cell Signaling Technology Inc., Danvers, MA, USA). Proteins were visualized with horseradish peroxidase- (HRP-) conjugated anti-rabbit IgG (1 : 2000; #7074, Cell Signaling Technology Inc., Danvers, MA, USA) or HRP-conjugated anti-mouse IgG (1 : 2000; #7076,Cell Signaling Technology Inc., Danvers, MA, USA), followed by use of the ECL chemiluminescence system (#6883, Cell Signaling Technology Inc., Danvers, MA, USA). The intensity of immunoreactive bands was analyzed and expressed as the ratio of PTEN, anti-phosphorylated Akt, Akt, and GTPCH I to *β*-actin protein in human EPCs.

### 2.6. Intracellular BH4 and NO Measurement

Intracellular BH4 concentrations were measured according to our previous report and another study [23, 33], by subtracting the level of BH2 plus oxidized biopterin from total biopterins, expressed as pmol/mg protein. The intracellular NO level in EPCs was evaluated by fluorescence microscopy using DAF-FM (Invitrogen) fluorescence as described previously [23] and expressed as a percentage change in fluorescence with respect to cells used as a time/vehicle control. The data were statistically compared relative to those of the static condition group.

### 2.7. Murine Hindlimb Ischemia Model

All animal care protocols and experiments were reviewed and approved by the Animal Care and Use Committee of the Laboratory Animal Research Center at Xiangya Medical School of Central South University. All of the mice were maintained in the specific pathogen-free facility of the Laboratory Animal Research Center at Central South University. Hindlimb ischemia was induced in 8-10-week-old male NRMInu/nu athymic nude mice. In brief, the mice were anesthetized with pentobarbital sodium (0.5%, 50 mg/kg) by intraperitoneal injection, and the surgical procedures were performed under sterile conditions. A vertical longitudinal incision was made in the left hindlimb, and the femoral artery and its branches were then dissected and ligated [18, 30, 34].

### 2.8. Laser Speckle Imaging

The blood flow was measured by scanning both rear paws with laser-speckle contrast imaging (PeriCam PSI Z, Perimed, Sweden) before and after the surgical procedure (days 0, 3, 7, 14, and 21). During the procedure, the animals were kept under pentobarbital sodium anesthesia and their body temperatures were strictly maintained between 36.5°C and 37.5°C [35]. Plantar perfusion was quantified within anatomically defined regions of interest (ROIs). The resulting data are reported as the ratio of blood flow in the left to right (L/R) hindlimb.

### 2.9. Immunohistochemistry

The gastrocnemius muscles were harvested at day 14 after femoral artery ligation. The midzone of the muscles (the 5 mm wide centermost section) was trimmed. The samples were embedded in paraffin, and 4 *μ*m thick cross-sections were made to prepare for hematoxylin and eosin (H&E) staining. The number of collateral arteries per field was measured under a microscope at 40x magnification. For immunohistochemistry, we used antibodies against CD31 (1 : 200, Abcam, UK). The paraffin section was rehydrated, and endogenous peroxidase activity was blocked for 30 min in methanol containing 0.3% hydrogen peroxide. The section was incubated with the primary antibody at 4°C overnight, followed by 60 min of incubation with the biotinylated secondary antibody (1 : 500, Abcam, UK) [36]. All specimens were counterstained with hematoxylin staining solution (Beyotime Institute of Biotechnology, China) and then sealed with neutral gum for storage. Analyses of morphology and degree of angiogenesis were performed after scanning the section through OLYMPUS CX41 and Leica Application Suite 4.0 software.

### 2.10. Statistical Analysis

All the data were statistically analyzed using GraphPad Prism 6.0 software with one-way analysis of variance for comparisons of three or more groups, followed by the least significant difference post hoc test. Values of *P* < 0.05 were considered statistically significant.

## 3. Results

### 3.1. Laminar Shear Stress Enhanced the Functions of Late EPCs *In Vitro*

**Figure 1:**
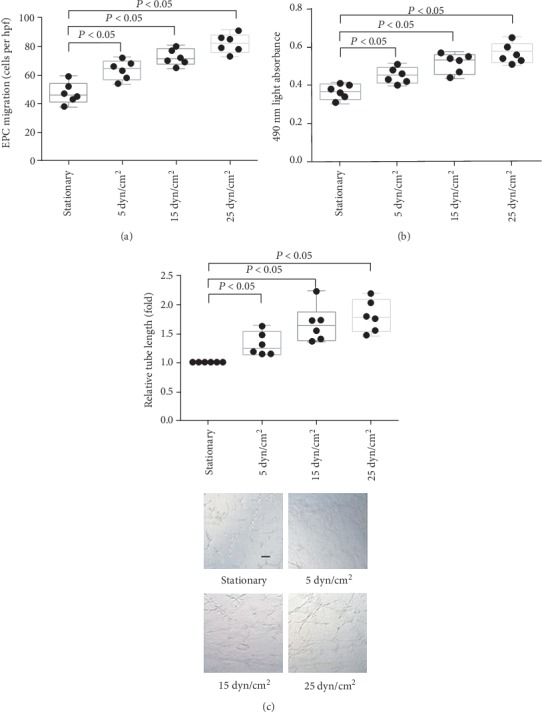
The effect of different levels of LSS on *in vitro* function of late EPCs. (a) Quantification analysis of VEGF-induced migration of late EPCs (^∗^*P* < 0.05 vs. EPCs under static condition group, *n* = 6 per group). (b) Quantification analysis of proliferative activity of late EPCs (^∗^*P* < 0.05 vs. EPCs under static condition group, *n* = 6 per group). (c) Quantification analysis and representative photograph tube information of late EPCs (represented as relative value to EPCs under static condition group, ^∗^*P* < 0.05 vs. EPCs under static condition group, *n* = 6 per group). Scale bar 200 *μ*m. Least significant difference was applied for the post hoc test in statistical analysis. hpf = high power field.

**Figure 2:**
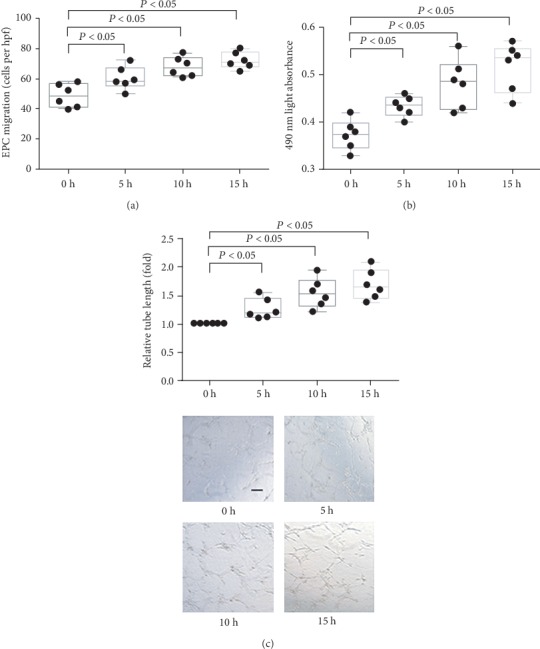
The effect of LSS on *in vitro* function of late EPCs at different time points. (a) Quantification analysis of VEGF-induced migration of late EPCs (^∗^*P* < 0.05 vs. EPCs at 0 h, *n* = 6 per group). (b) Quantification analysis of proliferative activity of late EPCs (^∗^*P* < 0.05 vs. EPCs at 0 h, *n* = 6 per group). (c) Quantification analysis and representative photograph tube information of late EPCs (represented as relative value to EPCs at 0 h, ^∗^*P* < 0.05 vs. EPCs at 0 h, *n* = 6 per group). Scale bar 200 *μ*m. Least significant difference was applied for the post hoc test in statistical analysis. hpf = high power field.

The migration (Figures 1(a) and 2(a)), proliferation (Figures 1(b) and 2(b)), and tube formation (Figures 1(c) and 2(c)) of EPCs increased after laminar shear stress in a dose-dependent and time-dependent manner.

### 3.2. Laminar Shear Stress Regulated the PTEN/Akt and GTPCH/BH4 Pathways in EPCs

**Figure 3:**
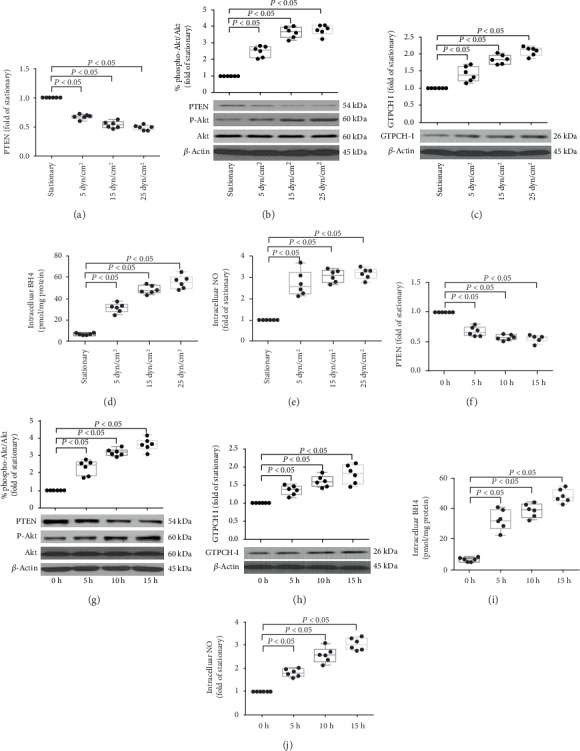
The effect of LSS on PTEN/Akt and GTPCHI/BH4 pathway in EPCs. (a, b) Representative photographs and quantification analysis of PTEN protein expression (a) and phosphorylation of Akt (b) of late EPCs treated with 5, 15, and 25 dyn/cm^2^ shear stress for 15 hours or under stationary condition, respectively (^∗^*P* < 0.05 vs. EPCs under static condition group, *n* = 6). (c) Representative photographs and quantification analysis of GTPCH I protein expression of late EPCs treated with 15 dyn/cm^2^ shear stress for 5, 10, and 15 hours (^∗^*P* < 0.05 vs. EPCs under static condition group, *n* = 6). (d, e) Quantitative analyses of intracellular BH4 (d) and NO (e) levels in late EPCs treated with 15 dyn/cm^2^ shear stress for 5, 10, and 15 hours, respectively (^∗^*P* < 0.05 vs. EPCs under static condition group, *n* = 6). (f, g) Representative photographs and quantification analysis of PTEN protein expression (f) and phosphorylation of Akt (g) of late EPCs treated with 5, 15, and 25 dyn/cm^2^ shear stress for 15 hours or under stationary condition, respectively (^∗^*P* < 0.05 vs. EPCs at 0 h, *n* = 6 per group). (h) Representative photographs and quantification analysis of GTPCH I protein expression of late EPCs treated with 15 dyn/cm^2^ shear stress for 5, 10, and 15 hours (^∗^*P* < 0.05 vs. EPCs at 0 h, *n* = 6 per group). (i, j) Quantitative analyses of intracellular BH4 (i) and NO (j) levels in late EPCs treated with 15 dyn/cm^2^ shear stress for 5, 10, and 15 hours, respectively (^∗^*P* < 0.05 vs. EPCs at 0 h, *n* = 6 per group). Least significant difference was applied for the post hoc test.

As shown in Figure 3, after laminar stress treatment, the PTEN expression level decreased and the Akt phosphorylation level increased in dose- and time-dependent manners. In addition, GTPCH I expression and the intracellular BH4 and NO levels in late EPCs were also dose- and time-dependently increased in response to laminar shear stress. Collectively, these data demonstrated that LSS decreased PTEN expression, induced Akt phosphorylation, and subsequently induced GTPCH/BH4 pathway activation in late EPCs.

### 3.3. Laminar Shear Stress Triggered PTEN/Akt Activation Upstream of the GTPCH/BH4 Pathway in Late EPCs

**Figure 4:**
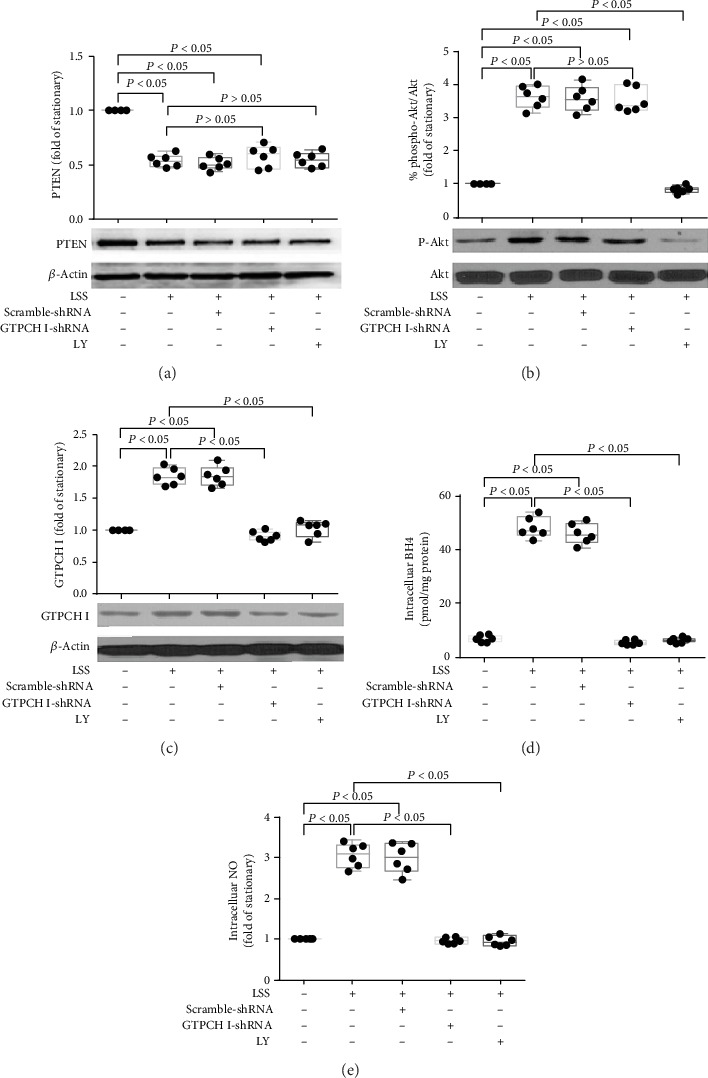
The relationship between PTEN/Akt and GTPCHI/BH4 pathway in EPCs in response to LSS. (a) Representative photograph and quantitative analysis of the effects of Akt inhibition or GTPCH I knockdown on LSS-regulated PTEN protein expression in late EPCs (^∗^*P* < 0.05 vs. EPCs under static condition group, *n* = 6; NS: no significant difference vs. LSS+EPC group, *n* = 6 per group). (b) Representative photograph and quantitative analysis of the effects of Akt inhibition or GTPCH I knockdown on LSS-regulated Akt phosphorylation of EPCs in late EPCs (^∗^*P* < 0.05 vs. EPCs under static condition group, *n* = 6; NS: no significant difference vs. LSS+EPC group, *n* = 6 per group). (c) Representative photograph and quantitative analysis of the effects of Akt inhibition or GTPCH I knockdown on LSS-regulated GTPCH I protein expression in late EPCs (^∗^*P* < 0.05 vs. EPCs under static condition group, *n* = 6; ^#^*P* < 0.05 vs. LSS+EPC group, *n* = 6 per group). (d, e) Quantitative analyses of the effects of Akt inhibition or GTPCH I knockdown on LSS-regulated intracellular BH4 (d) and NO (e) levels in late EPCs (^∗^*P* < 0.05 vs. EPCs under static condition group, *n* = 6; ^#^*P* < 0.05 vs. LSS+EPC group, *n* = 6 per group). Least significant difference was applied for the post hoc test in statistical analysis. Concentration of LY 294002: 10 *μ*mol/l. LSS = laminar shear stress.

As expected, pretreatment with GTPCH I shRNA did not prevent the laminar shear stress-induced reduction of PTEN expression and Akt phosphorylation elevation, and inhibition of Akt phosphorylation also did not change the expression level of PTEN under LSS (Figures 4(a) and 4(b)). In contrast, shRNA-mediated knockdown of GTPCH I significantly abolished the LSS-induced GTPCH I expression, leading to reductions in both the intracellular BH4 and NO levels in late EPCs. The same effects were observed after treatment of the Akt phosphorylation-specific inhibitor LY (Figures 4(c)–4(e)). These results demonstrated that laminar shear stress activates the GTPCH/BH4 pathway through suppressing PTEN expression and inducing Akt phosphorylation; in other words, PTEN/Akt acts as upstream of GTPCH/BH4 in the regulation of LSS on EPCs.

### 3.4. The PTEN/Akt/GTPCH/BH4 Pathway Mediated Shear Stress-Enhanced *In Vitro* Function and *In Vivo* Angiogenesis Capacity of Late EPCs

**Figure 5:**
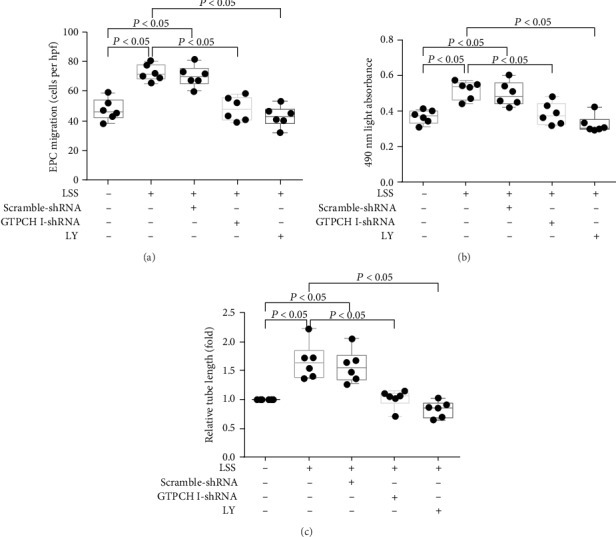
PTEN/Akt and GTPCH/BH4 pathway blockade inhibits *in vitro* function of EPCs treated with LSS. Quantification analysis of migration (a), proliferative activity (b), and tube formation (represented as relative value to EPCs under static condition group) of late EPCs (c) of EPCs treated with GTPCH I knockdown and Akt inhibition (^∗^*P* < 0.05 vs. EPCs under static condition group, *n* = 6; ^#^*P* < 0.05 vs. LSS+EPC group, *n* = 6 per group). Least significant difference was applied for the post hoc test in statistical analysis. Concentration of LY 294002: 10 *μ*mol/l. LSS = laminar shear stress.

**Figure 6:**
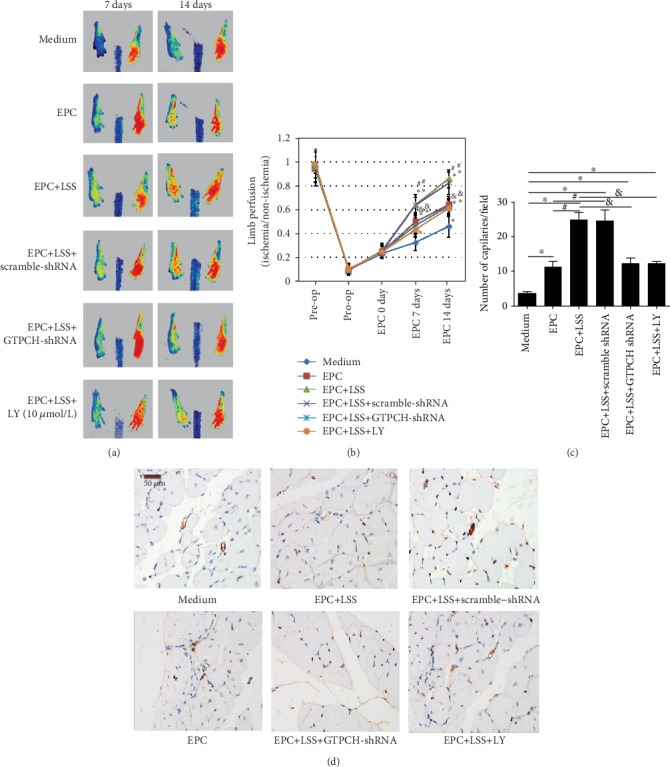
PTEN/Akt and GTPCH/BH4 pathway blockade inhibits *in vivo* angiogenesis of EPCs treated with LSS. Representative photographs (a) and quantification analysis showing *in vivo* angiogenesis capacity of EPCs treated with GTPCH I knockdown and Akt inhibition for improving blood perfusion in ischemic hind limb (b, c) (^∗^*P* < 0.05 vs. medium, *n* = 6; ^#^*P* < 0.05 vs. EPCs under static condition group, *n* = 6; ^&^*P* < 0.05 vs. LSS+EPC group, *n* = 6 per group). Least significant difference was applied for the post hoc test in statistical analysis. Concentration of LY 294002: 10 *μ*mol/l. (d) Representative photographs and quantification analysis showing *in vivo* angiogenesis capacity of EPCs by CD31 staining (^∗^*P* < 0.05 vs. medium, *n* = 6; ^#^*P* < 0.05 vs. EPCs under static condition group, *n* = 6; ^&^*P* < 0.05 vs. LSS+EPC group, *n* = 6 per group). Scale bar 50 *μ*m. Concentration of LY 294002: 10 *μ*mol/l. LSS = laminar shear stress; hpf = high power field.

Then, we evaluated the role of the PTEN/Akt/GTPCH/BH4 pathway in EPC function. Pretreatment with GTPCH I shRNA or LY attenuated the laminar shear stress-induced migration, proliferation, and tube formation activity of late EPCs (Figure 5). Consistent with the *in vitro* results, laminar shear stress remarkably improved the functional recovery of limb ischemia after femoral artery ligation in the mouse model (Figure 6). Moreover, this functional recovery was abolished by GTPCH I shRNA or LY.

Finally, CD31 staining of the gastrocnemius muscles on the 14th day after femoral artery ligation showed that LSS significantly enhanced angiogenesis in the ischemic limbs of mice, which was inhibited by GTPCH I shRNA or LY (Figure 6(d)). Collectively, these results confirmed that LSS triggered late EPC function both *in vivo* and *in vitro* via the PTEN/Akt/GTPCH/BH4 pathway.

## 4. Discussion

In this study, we observed that LSS downregulated PTEN expression, induced Akt phosphorylation, activated the GTPCH/BH4 signaling pathway, and elevated the *in vitro* activities and angiogenesis ability of late EPCs. Thus, LSS triggered late EPC-associated angiogenesis via the PTEN/Akt/GTPCH/BH4 pathway, revealing a novel mechanism underlying the beneficial effect of LSS on late EPCs. This indicates that shear stress can act as a potential therapeutic option for ischemic diseases.

EPCs derived from the bone marrow play pivotal roles in promoting neovascularization, repairing endothelial damage, and improving endothelial function [37–39]. Both EPC number and function are impaired in cardiovascular disease and associated risk factors [40–42], constituting an important mechanism of vascular injury. Previous studies further revealed that late EPCs exhibit high angiogenic potential and enhanced neovascularization in critical limb ischemia or myocardial infarction via directly engrafting into newly formed host vessels and promoting paracrine function [3, 43]. Accordingly, the angiogenesis capacity of late EPCs plays a fundamental role in the pathophysiology of vascular injury in ischemic diseases, thus showing promise as an effective therapeutic approach for ischemic diseases.

Accumulating data suggest that shear stress modulates the morphology, functions, and expression of functional genes in ECs [44, 45], thus emerging as an important nonpharmacological approach for vascular repair. We previously reported that LSS increased the proliferative and migratory activities and enhanced the repair ability of early EPCs in the injured endothelium by upregulating the expression of eNOS, Tie2, and GTPCH I [18, 20–23, 46]. Shear stress was also found to regulate the differentiation of late EPCs via upregulation of integrins or cytoskeletal rearrangement [47, 48]. However, the effect of LSS on the late EPC-mediated angiogenesis capacity has remained unclear. The present study demonstrated that LSS enhanced the proliferation, migration, and tube formation ability of late EPCs *in vitro*. Furthermore, 15 dyn/cm^2^ LSS treatment for 15 h significantly increased the angiogenesis of late EPCs in a murine hindlimb ischemic model. Thus, LSS upregulates the angiogenesis process mediated by late EPCs, further supporting its beneficial effect on EPC function and shedding light on the potential application of LSS as a nonpharmacological treatment option in ischemic disease. Combined with the research results showing that LSS could induce restoration of in vitro migration, proliferation, and adhesion of early EPCs by upregulating GTPCH/BH4 pathway in our previous research, we come to conclusion that LSS could stimulate both the *in vitro* function of early EPCs and late EPCs through GTPCH/BH4 pathway and improve their *in vivo* reendothelialization or angiogenesis function, respectively (since their different fundamental physiological functions *in vivo*).

PTEN participates in the angiogenic function of ECs as well as postnatal neovascularization [24, 49] and acts on EPC-mediated angiogenesis via PI3K/Akt/eNOS [26], which performed as increased PTEN suppresses PI3K and Akt activation, and inhibited PTEN expression gets the opposite effect. A previous study revealed that the GTPCH/BH4 pathway is associated with the early EPC-mediated endothelial repair capacity in hypertension [50], and we also found that LSS ameliorates the reendothelialization capacity of early EPCs by upregulating the GTPCH/BH4 pathway [23], confirming the essential role of this pathway in maintaining early EPC function. Moreover, the expression and activity of GTPCH I and biosynthesis of BH4 are stimulated under the condition of PTEN downregulation and subsequent Akt activation in late EPCs, thus enhancing their regenerative function [29]. Accordingly, we speculated that LSS affects the functional activity of late EPCs through the PTEN/Akt/GTPCH/BH4 pathway, which was supported by the fact that LSS downregulated PTEN expression, accelerated Akt phosphorylation, and increased GTPCH and BH4 levels in late EPCs. This effect was diminished by inhibiting Akt phosphorylation, indicating that GTPCH/BH4 acts downstream of the PTEN/Akt pathway of late EPCs in response to shear stress. This was further confirmed by the suppression of LSS-induced enhancement of BH4 and intracellular NO levels by GTPCH I knockdown; however, GTPCH I did not affect PTEN expression or Akt phosphorylation in late EPCs. Furthermore, inhibition of Akt and blockage of the GTPCH/BH4 pathway attenuated the LSS-induced increased *in vitro* activities and the *in vivo* neovascularization capacity or vascular density of late EPCs. Thus, these results highlight the importance of the PTEN/Akt and GTPCH/BH4 pathways in the LSS-mediated regulation of EPC *in vitro* function and *in vivo* neovascularization capacity.

Our previous study revealed that LSS can augment eNOS mRNA expression [20], activate the Tie2/Akt/eNOS signaling pathway [18], and upregulate the GTPCH/BH4 pathway [23] in early EPCs with subsequent enhancement of their endothelial repair capacity, indicating that LSS-stimulated NO production in early EPCs involves both posttranscriptional (including mRNA expression and subsequent phosphorylation of functional proteins involved in the NO production) and transcriptional modulation. It presented that LSS enhanced the phosphorylation of Tie2 and Akt, and Tie2 knockdown or Akt inhibition suppressed LSS-induced phosphorylation of Akt and eNOS in EPCs, which subsequently inhibited reendothelialization capacity of EPCs [18]. Moreover, it was found that LSS restored the Janus kinase 2 (JAK2) phosphorylation via upregulation of SDF-1/CXCR4 and then stimulated EPC-mediated reendothelialization. By contrary, CXCR4 knockdown by sh-RNA or JAK2 inhibition diminished this beneficial effect [30]. Other investigations demonstrated the roles of CXCR4 and CXCR7 in EPC homing and angiogenesis, through inhibition of SDF-1 inducing EPC activities by blocking CXCR4 or CXCR7 with their antibodies or antagonists [51], which showed an effect of depression on EPC migration, adhesion, and tube formation by both CXCR4 or CXCR7 inhibition. Among those researches, it was also suggested that Akt acts as a downstream signaling of angiopoietin-2/Tie2 [18] and CXCR7/SDF-1 pathway [51, 52] and regulates both *in vitro* functions and *in vivo* reendothelialization capacity of EPCs [30]. The present study further expounds that the LSS-induced increase in late EPC function and angiogenesis capacity is related to Akt and the GTPCH/BH4 pathway, suggesting that posttranscriptional regulation may play a crucial role in LSS-induced NO biosynthesis in late EPCs. Consistent with our previous research [23], it verified that eNOS/NO is the downstream signaling of GTPCH/BH4 pathway of EPCs. Therefore, modulation of the NO production of EPCs by LSS may be mediated by multiple mechanisms, and NO-dependent pathway is probably the common downstream target in modulating early or late EPC functions.

There are certain limitations of our study that should be acknowledged. First, we did not conduct a gene transfection experiment of the negative regulatory factor PTEN to confirm its role in the upregulation of EPC function mediated by shear stress. Second, fluorescence tracing of EPCs was not performed in this study. Previous studies used fluorescence tracing to demonstrate that after EPC injection, late EPCs reside in the capillaries of the ischemic muscle and differentiate into endothelial cells to significantly improve blood flow recovery [23, 53, 54]. This evidence reveals the fate of late EPCs in EPC-mediated neovascularization in response to ischemia. Third, because we did not have access to an oscillatory flow apparatus, we were unable to investigate the effect of oscillatory shear stress on late EPCs. Previous studies demonstrated that the effect of oscillatory shear on the endothelium is associated with an atheroprone endothelial phenotype, which is distinct from the atheroprotective potential exerted by laminar shear stress [55–57]. Therefore, it is possible that oscillatory shear stress inhibits the functional activity of late EPCs, thereby opposing the effects of laminar shear stress-mediated regulation; however, further investigation is needed to investigate this possibility.

## 5. Conclusion

In conclusion, the current study is the first to demonstrate that shear stress enhanced the *in vitro* function and *in vivo* neovascularization capacity of late EPCs via the PTEN/Akt/GTPCH/BH4 pathway. This investigation provides novel insights into the protective effect of shear stress on EPC-mediated angiogenesis, suggesting shear stress as an important nonpharmacologic therapeutic strategy for ischemic diseases.

## Data Availability

The datasets used and/or analyzed during the current study are available from the corresponding authors on reasonable request.

## Abbreviations

EPCs: Endothelial progenitor cells
NO: Nitric oxide
eNOS: Endothelial nitric oxide synthase
PTEN: Tumor suppressor phosphatase and tensin homolog
PI3K: Phosphatidylinositol 3′-kinase
GTPCH I: GTP cyclohydrolase I
BH4: Tetrahydrobiopterin
LSS: Laminar shear stress.
